# A hyperspectral plant health monitoring system for space crop production

**DOI:** 10.3389/fpls.2023.1133505

**Published:** 2023-07-04

**Authors:** Jianwei Qin, Oscar Monje, Matthew R. Nugent, Joshua R. Finn, Aubrie E. O’Rourke, Kristine D. Wilson, Ralph F. Fritsche, Insuck Baek, Diane E. Chan, Moon S. Kim

**Affiliations:** ^1^Environmental Microbial and Food Safety Laboratory, Agricultural Research Service, U.S. Department of Agriculture, Beltsville, MD, United States; ^2^Amentum, NASA Kennedy Space Center, Merritt Island, FL, United States; ^3^Exploration Research and Technology, NASA Kennedy Space Center, Merritt Island, FL, United States

**Keywords:** hyperspectral imaging, reflectance, fluorescence, machine learning, plant stress

## Abstract

Compact and automated sensing systems are needed to monitor plant health for NASA’s controlled-environment space crop production. A new hyperspectral system was designed for early detection of plant stresses using both reflectance and fluorescence imaging in visible and near-infrared (VNIR) wavelength range (400–1000 nm). The prototype system mainly includes two LED line lights providing VNIR broadband and UV-A (365 nm) light for reflectance and fluorescence measurement, respectively, a line-scan hyperspectral camera, and a linear motorized stage with a travel range of 80 cm. In an overhead sensor-to-sample arrangement, the stage translates the lights and camera over the plants to acquire reflectance and fluorescence images in sequence during one cycle of line-scan imaging. System software was developed using LabVIEW to realize hardware parameterization, data transfer, and automated imaging functions. The imaging unit was installed in a plant growth chamber at NASA Kennedy Space Center for health monitoring studies for pick-and-eat salad crops. A preliminary experiment was conducted to detect plant drought stress for twelve Dragoon lettuce samples, of which half were well-watered and half were under-watered while growing. A machine learning method using an optimized discriminant classifier based on VNIR reflectance spectra generated classification accuracies over 90% for the first four days of the stress treatment, showing great potential for early detection of the drought stress on lettuce leaves before any visible symptoms and size differences were evident. The system is promising to provide useful information for optimization of growth environment and early mitigation of stresses in space crop production.

## 1 Introduction

In fresh produce production systems to be deployed in future NASA spacecraft, monitoring plant growth and health during crop development from seedling to harvest is needed to ensure the food safety and security of pick-and-eat salad crops consumed by astronauts during cis-lunar, lunar, and Martian missions (Monje et al., 2019). Currently, plant monitoring in growth chambers onboard the International Space Station (e.g., NASA’s Veggie and Advanced Plant Habitat) is conducted by estimating plant growth rates based on photographic analysis of daily increments in leaf area. This limited approach cannot detect plant stresses, nutrient deficiencies, and diseases, which usually develop days before any visible leaf changes are observed (Massa et al., 2016; Zeidler et al., 2019). There is a need for novel sensing techniques that can monitor plant health before visible symptoms appear. Compact and automated sensing systems that require minimal crew intervention are preferred for better fit in volume-limited plant growth chambers. Early stress detection ensures the food safety of the crops produced for human consumption.

Nondestructive optical sensing methods and imaging-based technologies have rapidly progressed and been adopted for plant phenotyping (Zhao et al., 2019; Yang et al., 2020). High-throughput phenotyping (HTP) platforms have been developed by public and private sectors for use under both natural field conditions and controlled environments in various applications. The field-based HTP platforms may use aerial-based or ground-based approaches for crop phenotyping. The aerial-based systems typically carry miniaturized and lightweight airborne sensors on unmanned aerial vehicles (UAVs), such as multi-rotor, fixed wing, and flying-wing UAVs as well as helicopters and blimps (Yang et al., 2017). The flight heights and speeds of UAVs can enable crop canopy imaging at field level to cover an entire plot within minutes. The ground-based systems have been built based on manually pushed carts (White & Conley, 2013), self-propelled tractors (Jiang et al., 2018), and overhead systems supported by gantry (Virlet et al., 2017) or cable (Bai et al., 2019). These systems can implement multiple sensing modalities for long-exposure measurements at plant level owing to their high sensor payloads and battery capacities. Crop phenotyping can also be carried out at leaf level using portable sensing devices such as a handheld hyperspectral imager (Wang et al., 2020). The measurements of individual leaves can be combined with geo-location information to create geo-referenced data for mapping plant health in the field. On the other hand, the HTP platforms for controlled-environment agriculture have been developed mainly for use in greenhouses and growth chambers, and can collect high quality phenotypic data from plants with low variability due to relatively uniform environments. Such collection is usually limited and difficult under natural field conditions. Large-scale commercial phenotyping systems designed for the greenhouses usually use plant-to-sensor approaches for automated inspection of plants on conveyor belts viewed from the side or overhead with multiple integrated imaging sensors (Ge et al., 2016). For the growth chambers, the phenotyping systems are generally configured in a sensor-to-plant setup to inspect the plant samples from above (Lien et al., 2019). The sensors can also be installed on robotic arms (e.g., eye-in-hand cameras) to conduct indoor phenotyping for individual plant samples (Bao et al., 2018).

Various imaging modalities have been developed for evaluating plant characteristics, such as growth, stress, and disease. Examples include color imaging for morphology and geometry inspection, near-infrared imaging for leaf water content assessment, fluorescence imaging for chlorophyll content evaluation, hyperspectral imaging for stress monitoring, thermal imaging for leaf temperature measurement, and 3D imaging for shoot and canopy structure profiling (Li et al., 2014). Hyperspectral imaging can simultaneously obtain both spectral and spatial information from a target, which makes it a powerful tool for many food and agricultural applications (Qin et al., 2017). Under controlled indoor environments, close-range hyperspectral reflectance imaging commonly uses halogen lamps for broadband illumination of plants and has been applied to plant phenotyping (Mishra et al., 2017). A variety of hyperspectral image analysis methods have shown promising results for detecting early onset of plant stress and disease (Lowe et al., 2017). Fluorescence imaging is another useful technique for plant monitoring applications, such as evaluation of plant resistance to pathogens (Rousseau et al., 2013) and detection of plant diseases (Pérez-Bueno et al., 2016). Recently, 3D imaging techniques have been combined with close-range hyperspectral imaging to reduce geometry effects of various plant structures on the spectral and spatial data, such as using a line laser scanner based on triangulation principle (Behmann et al., 2016) and a depth sensor based on time-of-flight principle (Huang et al., 2018). Integrating multimodal imaging modalities generally can enhance the sensing capabilities for plant phenotyping (Jiang et al., 2018; Bai et al., 2019; Nguyen et al., 2023). However, few sensing systems have been reported on combining reflectance and fluorescence imaging techniques into one system for *in situ* plant monitoring applications.

Traditional hyperspectral imaging systems based on separated spectrographs and cameras are bulky and heavy, difficult to move frequently or to fit into small spaces. Existing commercial greenhouse phenotyping systems are generally too large as well. Also, the high-power halogen lights commonly used for hyperspectral reflectance imaging generate a lot of heat, which is not ideal for illuminating tender plants at close range. Recently, all-in-one small hyperspectral cameras have been introduced to the market, which made it possible to develop compact imaging systems. Examples include a handheld hyperspectral camera with an integrated scanner for plant disease detection (Behmann et al., 2018), a snapshot hyperspectral camera on a microscope for bacteria identification in dairy products (Unger et al., 2022), and a miniature line-scan hyperspectral camera on a UAV for maize phenotyping (Nguyen et al., 2023). With the advancement of the LED technologies, broadband LED lights can provide an alternative for the traditional halogen lights for the reflectance measurement, which is particularly important when sample heating is a concern, such as in biomedical applications (Stergar et al., 2022). To develop next-generation hyperspectral imaging systems able to autonomously monitor plant health and food safety in future manned space missions, an interagency agreement has been established between USDA Agricultural Research Service (ARS) and NASA Kennedy Space Center (KSC) to leverage their complementary areas of expertise—ARS sensing technology development and KSC space crop production. As a first step of this collaborative research, this study aimed to develop a compact and automated hyperspectral prototype system for installation in a KSC plant growth chamber to conduct imaging experiments on pick-and-eat salad crops grown under controlled environments. The system was designed to collect both hyperspectral reflectance and fluorescence images within a single imaging cycle using broadband and UV-A LED lights. Specific objectives of this methodology paper were to (1) present the system design, development, software, calibration, operation, and hyperspectral image processing methods for plant samples and (2) demonstrate the system’s performance and capability with an example application for detecting drought stress for lettuce.

## 2 Materials and methods

### 2.1 Hyperspectral plant health monitoring system

#### 2.1.1 System development

**Figure 1:**
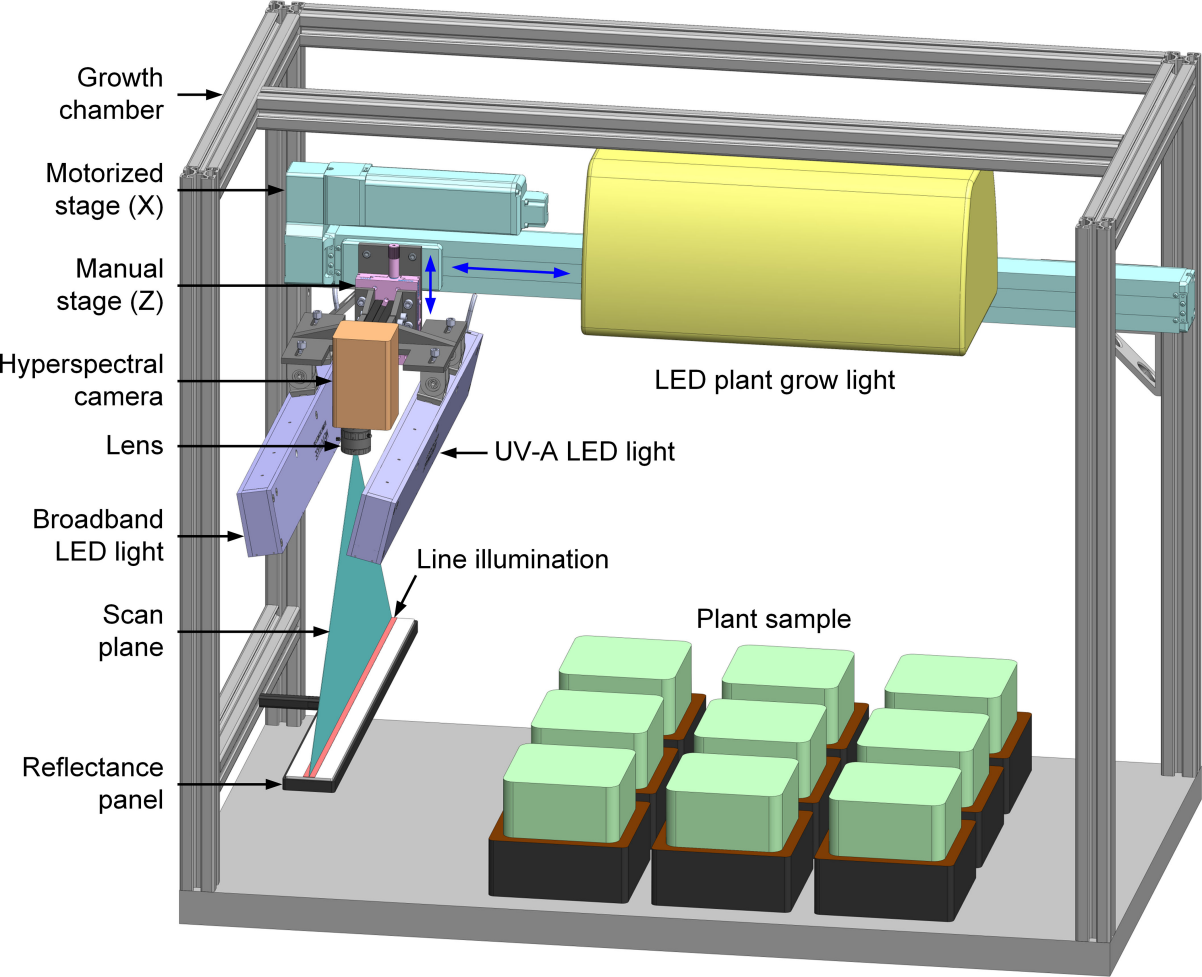
A compact and automated hyperspectral reflectance and fluorescence imaging system for plant health monitoring in a controlled-environment growth chamber.

The hyperspectral imaging system developed for automated health inspection of plants grown in a controlled-environment chamber is schematically illustrated in **Figure 1**. Since the system is designed for the confined space of the growth chamber to perform frequent imaging passes of stationary plants, all major hardware components, including lighting, camera, and translation stage, must be compact and lightweight for an overhead imaging setup. This sensor-to-sample arrangement differs from our previously developed hyperspectral systems that generally image moving samples using stationary lights and cameras (sample-to-sensor) (Kim et al., 2011). The system uses two LED line lights to illuminate the plant samples for hyperspectral image acquisition—one to provide visible and near-infrared (VNIR) light for reflectance imaging and, separately, the other to provide ultraviolet-A (UV-A) excitation light for fluorescence imaging. Specifically, LEDs at five VNIR wavelengths (428, 650, 810, 850, and 915 nm) and one UV-A wavelength (365 nm) are used in the two lights (UX-CLL509-C428C650IR810IR850IR915-24Z-US2 and UX-CLL509-UV365-24Z-US2, Metaphase Technologies, Bristol, PA, USA). Intensities of the LEDs at the six wavelengths can be independently adjusted by two digital dimming controllers (three channels each). Each light is mounted using two pivot joints at an angle of approximately 10° from the vertical position and has a full rod focal lens to create a narrow and structured beam (approximately 54 cm long and 1.5 cm wide on sample surface) with high intensity and concentrated light for a narrow, linear field of view. Each of the two line lights are enclosed in aluminum housings with minimum heat generation during rapid image acquisition.

A compact line-scan hyperspectral camera (microHSI 410, Corning Specialty Materials, Keene, NH, USA) is used to measure light reflectance/fluorescence from the plant samples in the VNIR wavelength range (400–1000 nm). A miniature solid block Offner imaging spectrograph and a CMOS focal plane array detector (12-bit and 1936×1216 pixels) are integrated in a small-form-factor package for the all-in-one hyperspectral camera. A wide-angle low-distortion lens with 5 mm focal length (Edmund Optics, Barrington, NJ, USA) allows imaging coverage of the full width of the growth chamber (53.3 cm) within the height of the chamber. A long-pass (>400 nm) gelatin filter (Wratten 2A, Kodak, Rochester, NY, USA) in front of the lens removes the UV-A excitation source peak at 365 nm and thus second-order effects around 730 nm. The lights and camera are on a manual translation stage (Thorlabs, Newton, NJ, USA) to enable 5 cm vertical adjustment for imaging plants of different heights. The manual stage is mounted on a linear motorized stage with a stroke of 80 cm (Intelligent Actuator, Los Angeles, CA, USA) that translates the lights and camera for overhead line-scan image acquisition. A reflectance standard panel with a custom size of 50×5 cm^2^ (Labsphere, North Sutton, NH, USA) is mounted under the camera’s origin position at one end of the chamber for flat-field correction to the reflectance images of the plants. When imaging is not actively taking place, a ceiling-mounted LED plant grow light (RX30, Heliospectra, Gothenburg, Sweden) provides continuous, simulated full-spectrum sunlight for photosynthesis in the plants. The growth chamber equipped with the imaging system is placed in a dark room to avoid the influence of ambient light on both plant growth and imaging.

#### 2.1.2 System software

**Figure 2:**
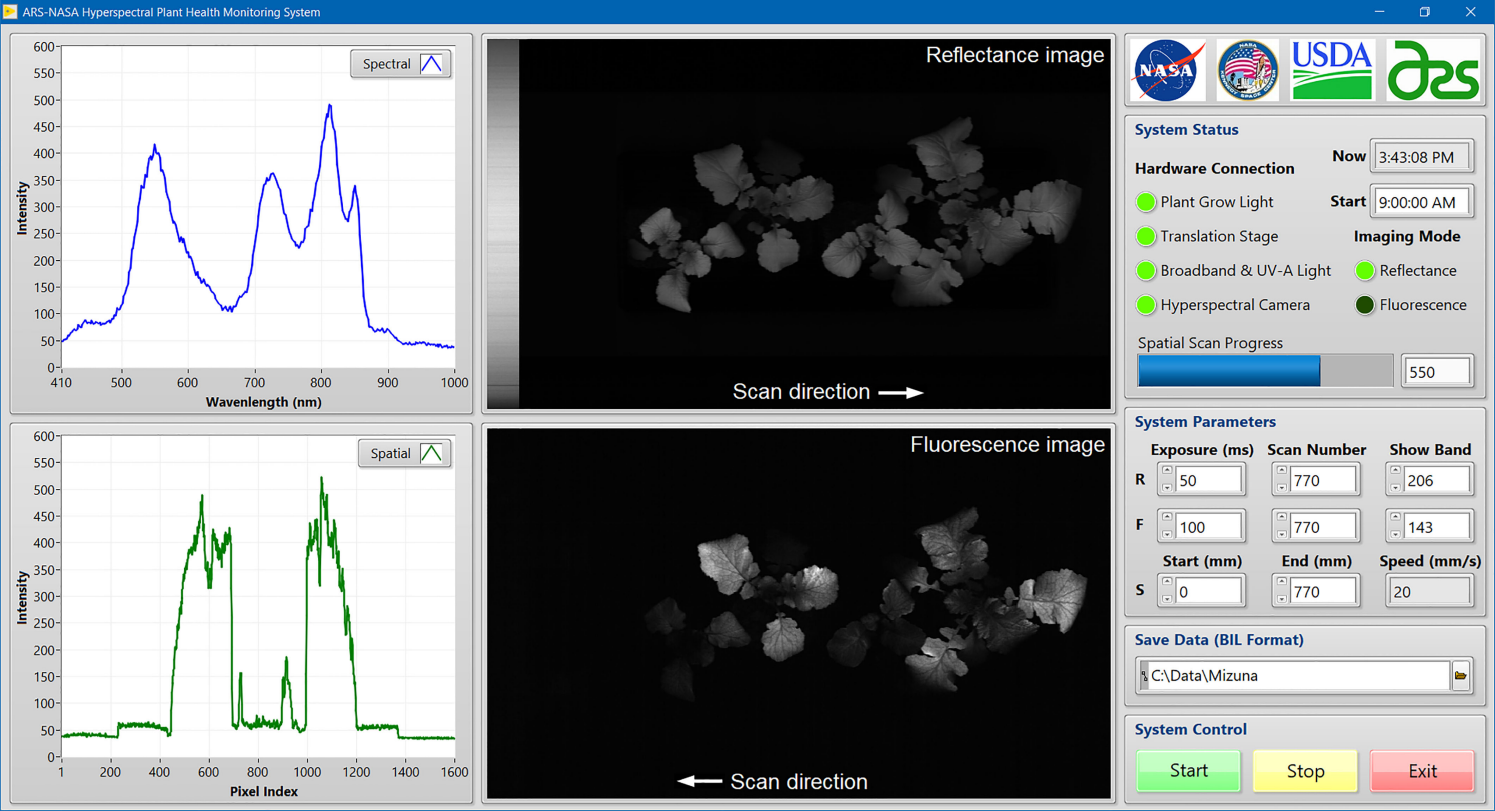
In-house developed LabVIEW software for system control and hyperspectral image acquisition from plant samples.

Imaging system software was developed using LabVIEW (v2017, National Instruments, Austin, TX, USA) in the Microsoft Windows 10 operating system on a laptop computer to provide a user-friendly graphic interface (**Figure 2**). Software development kits (SDKs) from the hardware manufacturers were used in the LabVIEW programming environment to communicate with major hardware components, including the VNIR and UV-A LED lights, the LED plant grow light, the hyperspectral camera, and the motorized translation stage. Functions from both the SDKs and LabVIEW were used to implement hardware parameterization and data transfer tasks, such as User Datagram Protocol (UDP) for LED light control, Universal Serial Bus (USB) for camera control, LabVIEW Vision Development Module (VDM) for image display and processing, and serial communication for stage movement control. Image acquisition can be started manually at any time or automatically using a timed imaging function (e.g., once a day at 9 AM). During image acquisition, a pair of reflectance and fluorescence images along with an original spectrum and a spatial profile are displayed and updated line by line to show the scan progress in real time. After each measurement, the reflectance and fluorescence images collected from the same scene are saved separately in a standard format of band interleaved by line (BIL) with timestamps appended to the filenames. The saved images can be processed and analyzed offline using in-house programs developed by MATLAB (R2022a, MathWorks, Natick, MA, USA).

#### 2.1.3 System operation

When an imaging cycle is initiated manually or automatically, the LED plant grow light is turned off to eliminate interference to the hyperspectral image acquisition. The VNIR line light is then turned on for 30 s to stabilize the LED output at five wavelengths. Then, the motorized translation stage begins moving towards the far end of the growth chamber. As the stage moves, the hyperspectral camera continuously collects line-scan reflectance signals while passing over the reflectance standard and then the plant samples below. When the sensing unit reaches the far end of the chamber, the reflectance image acquisition is completed and the VNIR light is turned off. Then, the UV-A line light is turned on and the camera begins continuous collection of line-scan fluorescence signals as the stage reverses movement back toward the origin position. When the stage reaches its original starting position, the UV-A light is turned off, completing one full imaging cycle that produces a pair of hyperspectral images, one reflectance and one fluorescence, for the same scene of the plant samples. Finally, the LED plant grow light is turned back on to continue providing simulated sunlight to the plants. In addition to the continuous moving mode, the system can also conduct incremental step-by-step hyperspectral scanning (i.e., stop-and-go mode), which generally does not need image registration to align the reflectance and fluorescence images of the same scene when identical step size is used for both imaging modes.

#### 2.1.4 System calibrations

**Figure 3:**
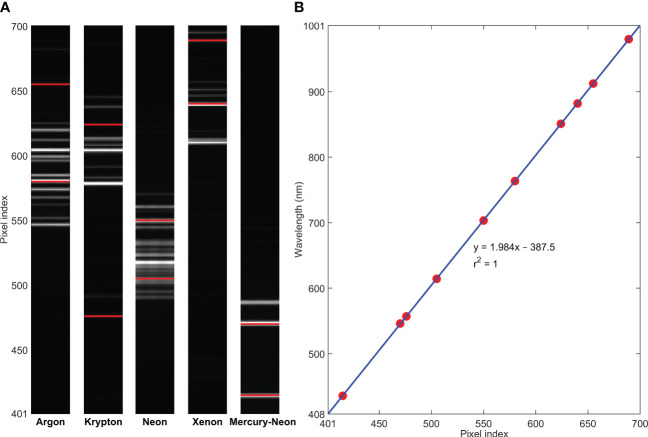
Spectral calibration for the hyperspectral camera using **(A)** standard pencil calibration lamps (two spectral lines selected from each lamp) and **(B)** a linear regression model.

Spectral calibration was conducted for the hyperspectral camera using five standard pencil calibration lamps, including argon, krypton, neon, xenon, and mercury-neon, to map pixel indices to wavelengths based on a linear regression model (**Figure 3**). For a total of 1216 pixels along the spectral dimension of the detector, it was found that pixel indices of 401–700 corresponded to the wavelength range of 408–1001 nm with an interval of 1.98 nm. Hence, only 300 pixels in the VNIR region are collected for the spectral acquisition. On the other hand, for spatial calibration of the system using a lens with 5 mm focal length and a working distance of 214 mm, the length of the instantaneous field of view (IFOV) of the camera was determined to be 484 mm across all 1936 spatial pixels, resulting in a spatial resolution of approximately 0.25 mm/pixel along the direction of the scanning line. For the moving direction of the imaging unit along a predetermined distance, the spatial resolution depends on the translation speed of the stage and the number of total scans. For example, using a moving speed of 20 mm/s, it will require 40 s to scan 800 lines for one-way travel over an 800-mm distance, resulting in an approximately 1 mm/pixel spatial resolution. To synchronize continuous line-scan image acquisition and translation stage movement, the moving speed of the stage is automatically determined by the system software based on the independently selected exposure time of the camera (i.e., low speed for long exposure time and high speed for short exposure time). Based on test results with the current laptop using selected exposure times and corresponding stage moving speeds, an empirical reciprocal relationship was found between the moving speed (V in mm/s) and the exposure time (T in s) (i.e., V=1/T). For example, for the exposure times of 0.05 and 0.1 s, the moving speeds were determined to be 20 and 10 mm/s, respectively. Note that this relationship may be affected by actual frame rate and USB data transfer speed when the camera is connected to different computers.

### 2.2 Hyperspectral image processing

This section describes procedures for processing raw hyperspectral reflectance and fluorescence images, including spectral and spatial averaging, flat-field correction, background removal, and image registration. Dragoon lettuce (*Lactuca sativa*) samples planted in 10 cm square soil pots were used to demonstrate the image processing results. Note that all images presented in this paper were acquired using the continuous scanning mode. The hyperspectral reflectance and fluorescence images were acquired from the standard panel and the lettuce in the pot using 0.05 s exposure time, 1200 spatial pixels, and 350 line scans over a 350 mm distance, which generated two 1200×350×300 (X×Y×λ) raw hypercubes. Initial smoothing of the spectral and spatial data was performed on the 3-D raw images by averaging across groups of three neighboring pixels in the spectral dimension (λ) and also across groups of four neighboring pixels in the camera’s scanning line direction (X), which created two 300×350×100 reduced hypercubes with a spatial resolution of 1 mm/pixel in both X and Y dimensions and a spectral interval of 5.94 nm. Using the VNIR reflectance values of the standard reference panel (i.e., 100%), flat-field correction (Kim et al., 2001) was conducted to convert the intensity values in the averaged image to relative reflectance values (0–100%).

To remove the background of the lettuce, correlation analysis was used to identify an optimal two-band reflectance ratio (i.e., Rλ1/Rλ2, where Rλn denotes single-band reflectance image at wavelength of λn) to segregate the leaves from the soil. Reflectance spectra of the lettuce and the soil were extracted from regions of interest (ROIs) that were manually selected from a single-band image at 815 nm (high reflectance for lettuce). The spectra of the lettuce and the soil were labeled with 1 and 2, respectively. Correlation coefficients were calculated between all two-band ratios of the ROI spectra and the label values. The ratio image that gave the highest correlation was converted to a binary mask image by a single threshold value computed using Otsu’s method (Otsu, 1979). To align the reflectance and fluorescence images of the same sample, a white paper printed with a grid of black dots was used as a calibration template for feature-based image registration with reflectance as a fixed image and fluorescence as a moving image. In-house programs developed by MATLAB were used to execute all the image processing procedures.

### 2.3 Application to plant drought stress detection

The hyperspectral plant health monitoring system is intended to be used for early detection of abiotic stresses (e.g., drought, overwatering, and nutrient deficiencies) and plant diseases (e.g., bacterial, fungal, and viral) for crops grown under controlled environments. A pilot study for detecting plant drought stress was conducted to demonstrate the performance and capability of the system, using Dragoon lettuce, a mini green romaine previously grown on the International Space Station. Each individual lettuce plant, of twelve total plants divided into two sets of six, was grown in mix of soil and arcillite (v:v=3:7) in a 10 cm square pot for a total of 28 days after seeding. The plants were cultivated under an air temperature of 23°C, a relative humidity of 65%, a CO_2_ concentration of 3000 ppm (simulating spacecraft cabin air), and a photoperiod of 16 h light and 8 h dark using a LED plant grow light providing a photosynthetic photon flux density (PPFD) of 300 µmol m^−2^ s^−1^ and a spectral output of 90% red, 1% green, and 9% blue. Note that in this experiment, the plant samples were grown under a LED grow light that was not in the imaging chamber. Two different moisture treatments for the soil were started on Day 12 after planting (i.e., stress Day 1) so that six lettuce samples were grown under well-watered conditions (control) and six were under-watered (drought stress). Each set of six pots was arranged in a 2×3 array on a tray placed inside the growth chamber. Moisture in each tray was controlled by an automated watering system that periodically watered the plants with nutrient solution to maintain a pre-determined volumetric moisture content (VMC), which was measured by a soil moisture sensor. The VMCs for the control and drought treatments were set as 50% and 30%, respectively. Thus, the average moisture content of the drought pots contained 100 ml less water than the controls. After introduction of the drought stress, hyperspectral images were taken over 13 days within a period of three weeks (i.e., Week 1: Days 1–4, Week 2: Days 7–11, and Week 3: Days 14–17). Reflectance and fluorescence images were collected from each tray using 1600 spatial pixels and 550 line scans across a 550 mm distance under a common camera exposure time of 0.05 s. It took approximately 90 s to obtain two 1600×550×300 raw hypercubes. Image processing was performed using the procedures described in Section 2.2, which generated two 300×360×100 reduced hypercubes for each set of six lettuce samples.

Three methods, including leaf area, band-ratio, and machine learning, were used for the drought stress detection. For all 13 sampling days in the three-week period, total leaf areas for each set of six samples were estimated daily by counting the pixel numbers in the plant mask images. Correlation analysis was then used to identify a two-band ratio for the drought stress detection. Reflectance spectra of control and drought lettuce samples were extracted from masked images for each day, which were then grouped into individual weeks for the correlation analysis. Last, the potential of machine learning method for early detection of drought stress was investigated using reflectance spectra of lettuce. A pixel average-window method was first used to remove the plant areas with large variations (e.g., leaf margins) and reduce the number of spectra used in machine learning classifications. All the lettuce pixels in the masked R815 images (high reflectance for lettuce) were grouped into 3×3 pixel windows, in which mean (M) and standard deviation (SD) of the pixel intensities were calculated. In each window, if there were more than 10% of nine pixels (i.e., one or more pixels) with the reflectance intensities beyond the range of M ± 3SD, the whole window was removed for further analysis. The nine spectra extracted from each remaining window were averaged in the spatial domain. All mean spectra labeled with control and drought were used for the machine learning classifications. Each of nine labeled datasets from stress Days 1 to 11 was input to the Classification Learner app in MATLAB, in which seven optimizable classifiers, including Naive Bayes, decision tree, ensemble, k-nearest neighbor (KNN), support vector machine (SVM), neural network (NN), and discriminant analysis, were used to compare the classification accuracies. Hyperparameter optimization functions within the app were used for automated selection of the hyperparameters for all the models to minimize the classification error. To simplify the evaluation of misclassification costs and model training and validation, equal penalty was assigned to all misclassifications. Accuracies of the seven classification models for the dataset on each stress day in the first two weeks were evaluated using a five-fold cross-validation method. To minimize variations from random dataset partitioning, training and validation of each model was repeated for ten times. The average cross-validation accuracy over the ten runs was used as the overall accuracy of each model.

## 3 Results and discussion

### 3.1 Hyperspectral image processing results

**Figure 4:**
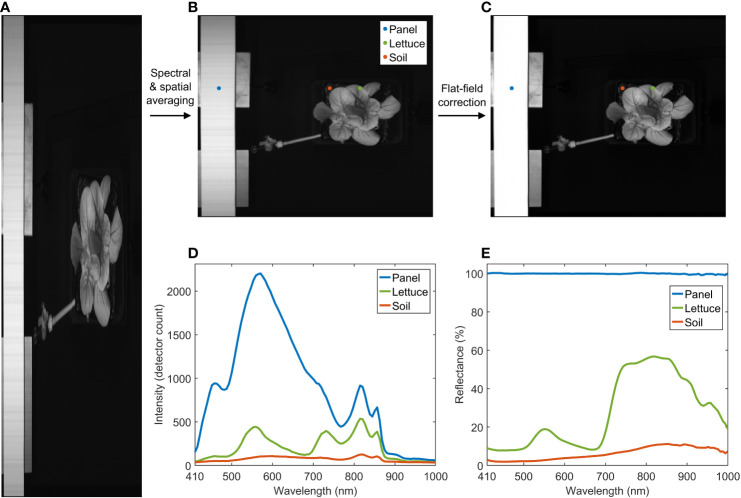
Spectral and spatial averaging and flat-field correction for hyperspectral reflectance image of a lettuce sample. **(A)** raw, **(B)** reduced, and **(C)** reflectance images at 815 nm, and **(D)** raw and **(E)** reflectance spectra of panel, lettuce, and soil.

**Figure 4** shows reflectance image processing results for a lettuce sample. After spectral and spatial averaging, a reduced hypercube (**Figure 4B**) was first generated from the raw hypercube (**Figure 4A**). Then, the reduced hypercube was converted to a reflectance hypercube (**Figure 4C**) *via* the flat-field correction. The averaged raw and reflectance spectra of the panel, the lettuce, and the soil at three selected locations are plotted in **Figures 4D, E**, respectively. The lettuce sample shows a typical vegetation reflectance spectrum in the VNIR region. The reflectance signals of the lettuce beyond 850 nm tend to drop and fluctuate toward 1000 nm due to relatively low output of the 915 nm LEDs, spectrograph‐produced second‐order effect (Kim et al., 2011), and a low quantum efficiency of the detector in this wavelength range.

**Figure 5:**
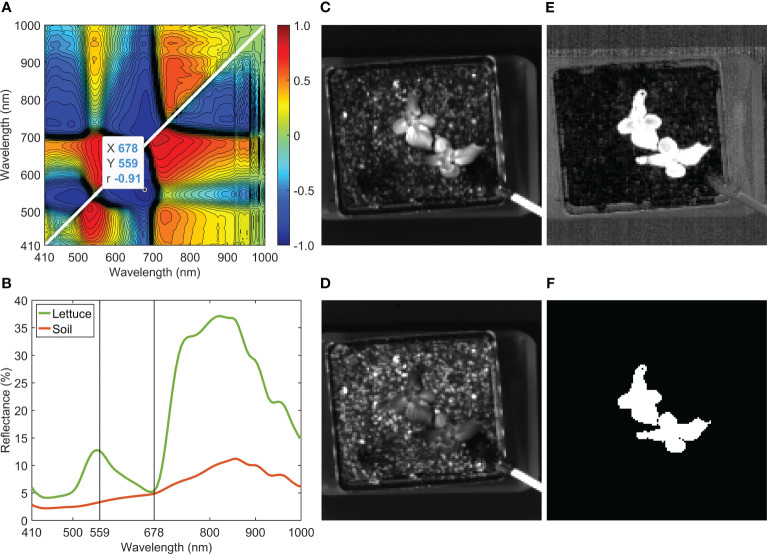
Background removal of a lettuce sample: **(A)** correlation analysis for band-ratio selection to segregate lettuce from soil, **(B)** mean reflectance spectra of lettuce and soil, reflectance images at **(C)** 559 nm and **(D)** 678 nm, **(E)** ratio image (R559/R678), and **(F)** mask image generated using R559/R678.

Removing the background of a lettuce sample in a soil pot is demonstrated in **Figure 5**. The hyperspectral reflectance image was collected and processed in the same way as those shown in **Figure 4**, but with the addition of image cropping to center and contain the whole plant pot in a reduced hypercube size of 130×130×100. The ratio between 559 and 678 nm (i.e., R559/R678) gave the maximum absolute correlation coefficient of −0.91 (**Figure 5A**). The selected two wavelengths are marked on top of the mean spectra of the lettuce and the soil (**Figure 5B**). Single-band images at 559 and 678 nm are shown in **Figures 5C, D**, respectively. The two mean spectra exhibit opposite trends between 559 and 678 nm, indicating that these wavelengths are effective selections for the correlation analysis and band-ratio method for background removal. As shown in **Figure 5E**, the contrast between the leaves and the background is greatly enhanced in the ratio image of R559/R678. Lastly, a mask image (**Figure 5F**) was obtained by applying a single threshold value to the ratio image. Note that although the optimal wavelength pair was selected based specifically on the reflectance spectra of the lettuce and the soil, the ratio of R559/R678 is also effective for removing other objects in the background, such as the pot, tray, and drip irrigation tubing.

**Figure 6:**
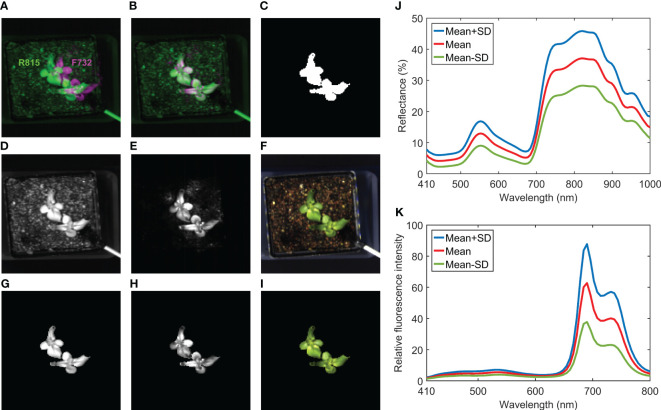
Image registration for hyperspectral reflectance and fluorescence images of a lettuce sample: overlap of **(A)** original and **(B)** registered R815 and F732 images, **(C)** mask image generated using R559/R678, **(D)** registered and **(G)** masked R815 images, **(E)** registered and **(H)** masked F732 images, **(F)** registered and **(I)** masked pseudo RGB images, and **(J)** reflectance and **(K)** fluorescence spectra extracted from masked images.

**Figure 6** illustrates image registration, masking, and spectral extraction for the hyperspectral reflectance and fluorescence images collected from the same lettuce sample shown in **Figure 5**. An overlap of R815 and F732 (i.e., single-band fluorescence image at 732 nm) images at their original spatial positions are shown in **Figure 6A**, which clearly illustrates misalignment of the reflectance and fluorescence images of the same scene. The registration result from the calibration template showed that a simple horizontal translation (10-pixel shift to the left for this case) is adequate to align the fluorescence image with the reflectance image. Thus, the double-image effect disappeared in the overlap of the registered R815 and F732 images (**Figure 6B**). Note that same camera exposure time of 0.05 s (thus same stage moving speed in continuous scanning mode) was used to acquire both reflectance and fluorescence images in this example. If different exposure times are used, then step-by-step scanning mode or more advanced image registration techniques may be needed to align the images. After the registration, the mask image created using R559/R678 (**Figure 6C**) can be used to mask both reflectance and fluorescence images (**Figures 6D, E, G, H**). In addition, a pseudo RGB image (registered and masked shown in **Figures 6F, I**, respectively) was generated using red, green, and blue bands of the reflectance hypercube to provide natural color and appearance for the lettuce sample. Based on the masked images, spectra can be extracted from all the lettuce pixels for further analysis. Mean and standard deviation (SD) reflectance and fluorescence spectra of the lettuce sample are plotted in **Figures 6J, K**, respectively. Due to chlorophyll a in the lettuce leaf tissue, the reflectance spectra show an absorption peak at 672 nm and the fluorescence spectra show two red emission peaks at 690 and 732 nm. In addition, owing to phenolic compounds in the leaves (Chappelle et al., 1991), two broad blue and green emission peaks with low fluorescence intensities were observed around 450 and 530 nm, respectively.

### 3.2 Plant drought stress detection results

#### 3.2.1 Leaf area results

**Figure 7:**
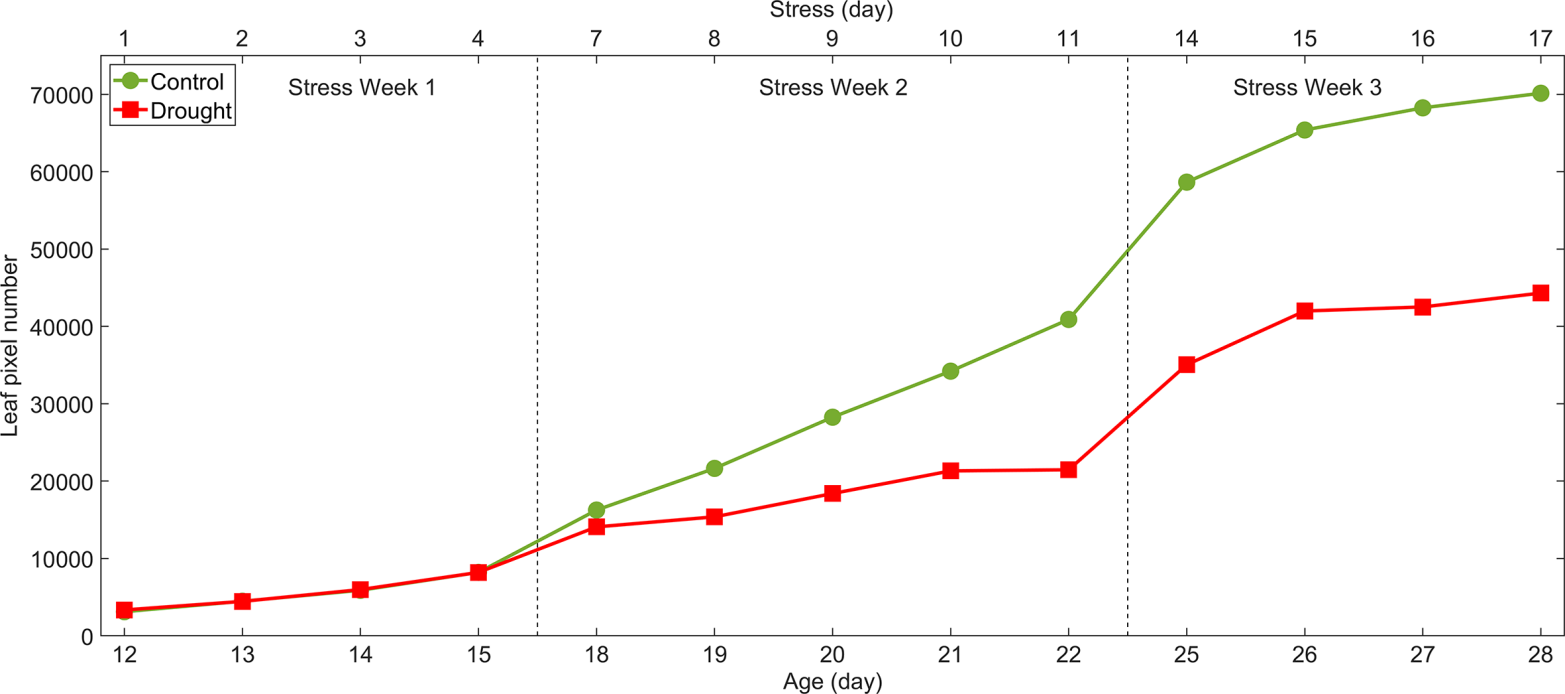
Leaf area method for detection of drought stress of lettuce: total leaf areas on each sampling day estimated by pixel numbers in mask images of control and drought lettuce samples.

Results for the leaf area method are plotted in **Figure 7**. The average pixel areas between control and drought plants on stress Days 3, 4, 7 and 8 were compared using a two-sample t-test. The pixel areas from drought plants on stress Days 3 and 4 were not significantly different from the controls. However, they were significantly different on stress Day 7 (13% less leaf area, p<0.02) and Day 8 (29% less leaf area, p<0.001). The decrease in leaf area caused by drought persisted until harvest.

#### 3.2.2 Band-ratio results

**Figure 8:**
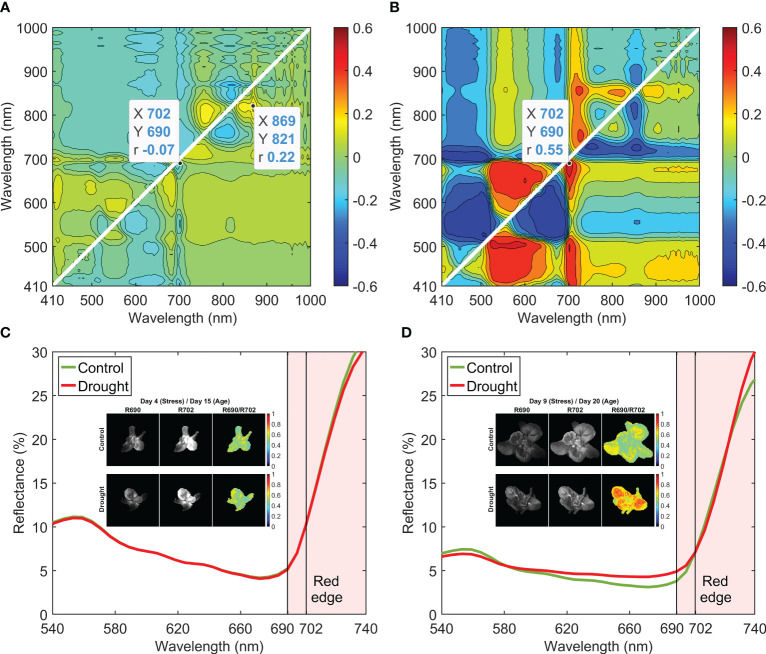
Correlation analysis for band-ratio selection to differentiate control and drought lettuce samples using reflectance spectra from **(A)** Week 1 (stress Days 1–4) and **(B)** Week 2 (stress Days 7–11). Normalized mean spectra and example single-band and band-ratio images for Weeks 1 and 2 are shown in **(C, D)**, respectively.

Correlation coefficients calculated using the reflectance spectra in Week 1 were low (**Figure 8A**), with a maximum of 0.22 at R821/R869. When Week 2 data were used, the maximum correlation increased to 0.55 at R690/R702 (**Figure 8B**). For each day in Week 2, the contour plot of correlation coefficients showed a similar pattern to that from using the data of the whole week. Mean reflectance spectra of the control and drought samples in Weeks 1 and 2 were normalized at 702 nm and are plotted across the wavelength range of 540–740 nm in **Figures 8C, D**, respectively. In Week 1, there was no notable spectral difference between 690 and 702 nm for the two moisture treatments, which is the reason for the low correlation coefficient (i.e., −0.07) at R690/R702 (**Figure 8A**). In Week 2, however, the mean spectrum of the drought samples showed higher reflectance at 690 nm than that of the control samples. Both selected wavelengths of 690 and 702 nm are in the red edge spectral region (690–740 nm), in which reflectance of the leaves is sensitive to the change of the chlorophyll content in green plants (Lowe et al., 2017). Single-band (R690 and R702) and band-ratio (R690/R702) images of a control and a drought lettuce samples on stress Days 4 and 9 are shown in **Figures 8C, D**, respectively. On Day 4, the two ratio images exhibited similar intensity patterns for the control and drought samples. On Day 9, however, the ratio image of the drought sample showed higher intensities over most of the leaf areas, in great contrast to the lower intensities of the control sample leaf areas.

**Figure 9:**
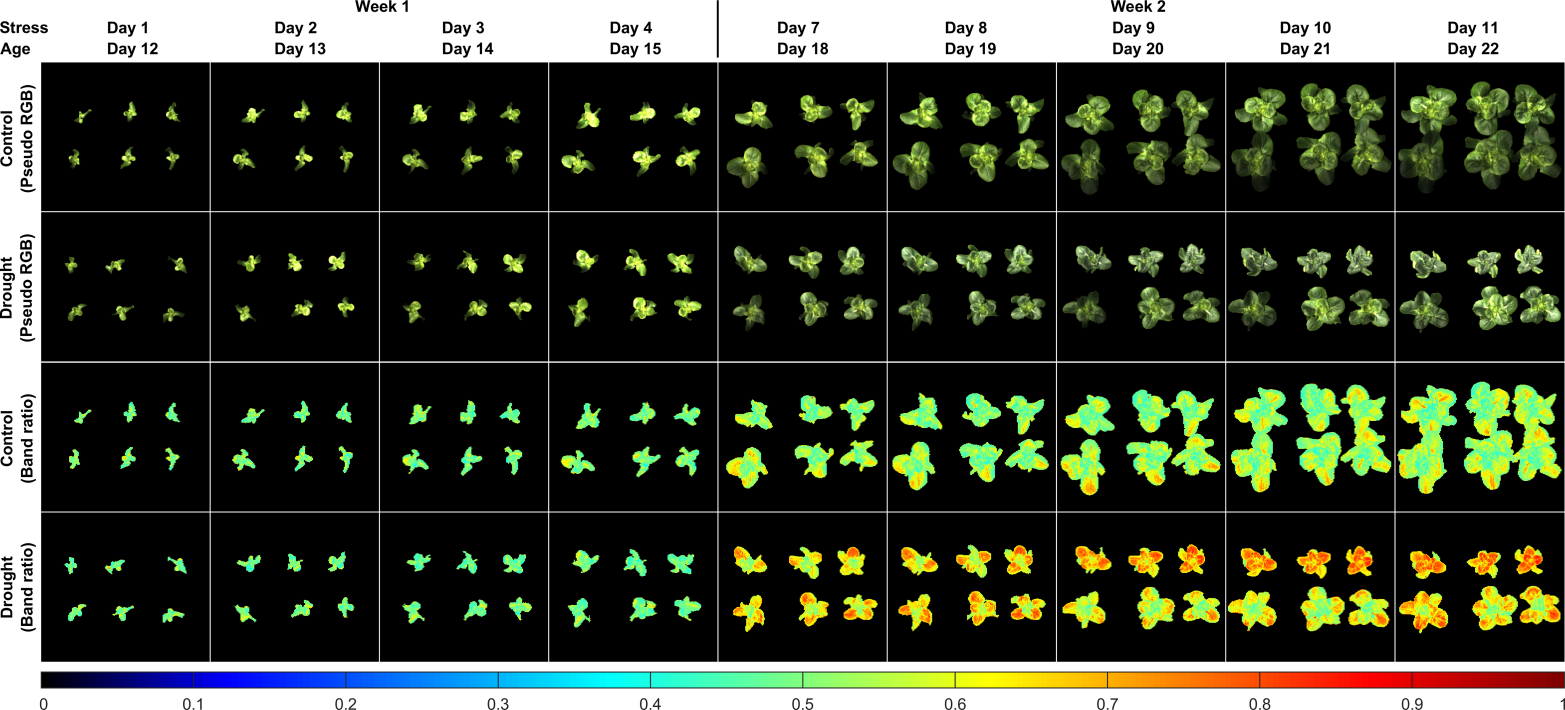
Reflectance band-ratio method for detection of drought stress of lettuce: pseudo RGB and ratio (R690/R702) images of samples grown under well-watered (control) and under-watered (drought) conditions in the first two sampling weeks.

**Figure 9** shows pseudo RGB and band-ratio images of all the lettuce samples in the first two weeks. The pseudo RGB images show that there were no apparent differences for the natural color and appearance of the lettuce grown under the two moisture treatments. For the ratio images from stress Days 1 to 4 in Week 1, no obvious intensity differences were observed between the control and drought samples. The first notable difference for the drought samples appeared in the ratio image on stress Day 7, in which all six plants showed higher ratio intensities than the controls. In Week 2, the ratio values of R690/R702 of the drought samples tended to increase over the whole leaf areas from stress Days 7 to 11, while those of the controls generally remained unchanged, except for some small leaf areas. From stress Days 9 to 11, the ratio images of the three drought samples on the bottom row showed lower intensities than those of the three on the top row. Such variations can probably be attributed to the non-uniform water supply received by each individual pot. As the drought samples were grown into stress Week 3, the ratio intensity variations of the six plants increased and their ratio intensity differences with the six control samples decreased (results not shown). These results suggest that the band-ratio method can detect the drought stress at approximately the same time as the leaf area method, which may not be adequate for the goal of early stress detection.

#### 3.2.3 Machine learning results

**Figure 10:**
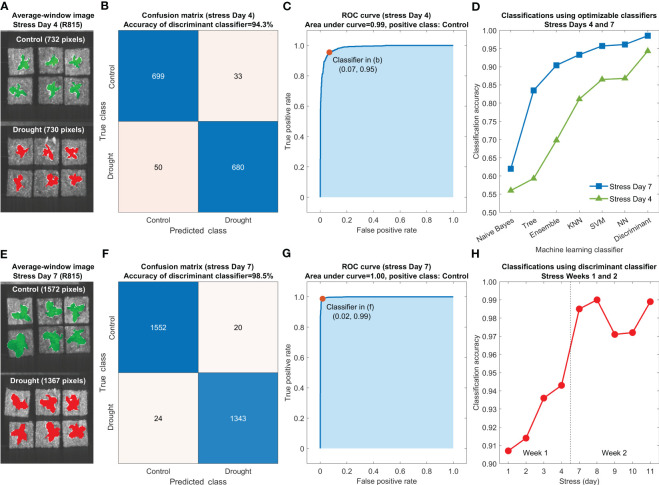
Machine learning classification method for early detection of drought stress of lettuce using reflectance spectra extracted from hyperspectral images of the leaves: average-window R815 images on stress Days **(A)** 4 and **(E)** 7, confusion matrices for stress Days **(B)** 4 and **(F)** 7 and ROC curves for stress Days **(C)** 4 and **(G)** 7 using discriminant classifiers, **(D)** classification accuracies using seven optimized classifiers for stress Days 4 and 7, and **(H)** classification accuracies using discriminant classifiers for all nine stress days in the first two weeks.

Accuracies for differentiating the control and drought lettuce samples using seven optimized classification models are shown in **Figure 10D** using the five-fold cross-validation results from stress Days 4 (**Figure 10A**) and 7 (**Figure 10E**). For both days, the Naive Bayes and the discriminant classifiers generated the lowest (worse than 65%) and the highest (better than 90%) accuracies, respectively, and the accuracies of other five classifiers fell between those two. Meanwhile, the accuracies of all seven classifiers using the reflectance spectra on Day 7 are consistently higher than those using the data on Day 4. Confusion matrices and receiver operating characteristic (ROC) curves on these two selected days using the discriminant classifier are shown in **Figures 10B, F** and **Figures 10C, G**, respectively. The classification accuracies for Days 4 and 7 are 94.3% and 98.5%, respectively, and the areas under both ROC curves are better than 0.98. Similar results were obtained for other days in the first two weeks. Since the discriminant classifier gave the best overall classification performance, it was selected for differentiating the control and drought lettuce samples in all nine test days in the first two weeks, and the results are summarized in **Figure 10H**. In Week 1, the classification accuracies gradually increased from Day 1 (90.7%) to Day 4 (94.3%). In Week 2, all the accuracies were higher than 97.0% with a maximum of 99.0% on Day 8. Results from this preliminary experiment suggest that the machine learning method using an optimized discriminant classifier based on VNIR hyperspectral reflectance images is promising for early detection of drought stress for lettuce without any visible symptoms and leaf size differences.

The results above demonstrate that the hyperspectral reflectance imaging based on the broadband LED light can be potentially used for plant health monitoring. The VNIR LED light provides an alternative to the halogen light commonly used for the reflectance measurement, which can avoid excessive heat from the halogen light projected to the tender plants under a close-range imaging setup. As the next step of this collaborative project, full-scale plant experiments with replications will be conducted to collect image data from salad crops of multiple species (e.g., lettuce, pak choi, mizuna, and radish) grown under different abiotic and biotic stress treatments. Both separate and combined use of the reflectance and fluorescence data for assessing plant vigor will be investigated and compared. Spectral and image fusion algorithms will be developed toward the goal of early detection of plant stresses and diseases. The hyperspectral plant health monitoring system has great potential to provide timely and useful information for optimization of the growth environment and early mitigation of plant stress and disease in space crop production systems, as well as for other applications in controlled-environment agriculture.

## 4 Conclusions

As a first fruit of a collaborative project between USDA ARS and NASA KSC, a compact hyperspectral imaging prototype system was developed and preliminarily tested for monitoring plant health in controlled-environment space crop production. The prototype system can acquire both hyperspectral reflectance and fluorescence images in the visible and near-infrared region within a single imaging cycle, which can provide rich spectral and spatial information to potentially carry out early detection of abiotic stresses and plant diseases in pick-and-eat salad crops. Compact and lightweight hardware components, including two LED line lights, a hyperspectral camera, and a motorized stage, were used to build the imaging unit to ensure it can be integrated into the confined space of a growth chamber to conduct overhead sensor-to-sample imaging. The broadband and UV-A LED lights project a narrow and structured beam to illuminate plant samples during rapid image acquisition. Use of VNIR LED light instead of traditional halogen light for reflectance can avoid excessive heat projected to the plant samples under a close-range imaging setup. The in-house developed system control software provides a user-friendly interface for plant scientists to conduct imaging experiments. The performance and capability of the developed system was demonstrated in a pilot study on plant drought stress detection for Dragoon lettuce. A reflectance band-ratio method based on two wavelengths selected in the red edge spectral region was found to be inadequate for early stress detection, as it could only differentiate control and drought samples at the same stage of growth as was possible from traditional leaf area estimation. A machine learning method using an optimized discriminant classifier based on VNIR reflectance spectra showed promise for early detection of drought stress on lettuce leaves lacking visible symptoms and size differences. To fully utilize the potential of hyperspectral reflectance and fluorescence imaging techniques to achieve the goal of detecting early onset of plant stresses and diseases in the space crop production, full-scale experiments on multiple species and treatments and the development of more advanced spectral and image analysis and fusion algorithms are planned as the next step of this project.

## Data availability statement

The original contributions presented in the study are included in the article/supplementary material. Further inquiries can be directed to the corresponding author.

## Author contributions

JQ: conceptualization, methodology, investigation, formal analysis, writing—original draft, writing—review and editing. OM: data curation, formal analysis, investigation, writing—review and editing. MN: validation, data curation. JF: validation, data curation. AO: data curation, investigation, writing—review and editing. KW: project administration, supervision, funding acquisition. RF: project administration, supervision, funding acquisition. IB: methodology, validation, investigation. DC: resources, writing—review and editing. MK: conceptualization, methodology, investigation, writing—review and editing, project administration, supervision, funding acquisition. All authors contributed to the article and approved the submitted version.
